# Protection of remote ischemic preconditioning against acute kidney injury: a systematic review and meta-analysis

**DOI:** 10.1186/s13054-016-1272-y

**Published:** 2016-04-20

**Authors:** Jiachang Hu, Shaopeng Liu, Ping Jia, Xialian Xu, Nana Song, Ting Zhang, Rongyi Chen, Xiaoqiang Ding

**Affiliations:** Division of Nephrology, Zhongshan Hospital, Fudan University, No. 180, Fenglin Road, Xuhui District, Shanghai, 200032 China; Shanghai Institute of Kidney and Dialysis, Shanghai, 200032 China; Shanghai Key Laboratory of Kidney and Blood Purification, Shanghai, 200032 China; Department of Nephrology, General Hospital of Ningxia Medical University, Ningxia, 750004 China

**Keywords:** Acute kidney injury, Cardiac surgery, Remote ischemic preconditioning, Renal replacement therapy, Percutaneous coronary intervention

## Abstract

**Background:**

Remote ischemic preconditioning (RIPC) is a promising approach to preventing acute kidney injury (AKI), but its efficacy is controversial.

**Methods:**

A systematic review of 30 randomized controlled trials was conducted to investigate the effects of RIPC on the incidence and outcomes of AKI. Random effects model meta-analyses and meta-regressions were used to generate summary estimates and explore sources of heterogeneity. The primary outcome was incidence of AKI and hospital mortality.

**Results:**

The total pooled incidence of AKI in the RIPC group was 11.5 %, significantly less than the 23.3 % incidence in the control group (*P* = 0.009). Subgroup analyses indicated that RIPC significantly reduced the incidence of AKI in the contrast-induced AKI (CI-AKI) subgroup from 13.5 % to 6.5 % (*P* = 0.000), but not in the ischemia/reperfusion-induced AKI (IR-AKI) subgroup (from 29.5 % to 24.7 %, *P* = 0.173). Random effects meta-regression indicated that RIPC tended to strengthen its renoprotective effect (*q* = 3.95, *df* = 1, *P* = 0.047) in these trials with a higher percentage of diabetes mellitus. RIPC had no significant effect on the incidence of stages 1–3 AKI or renal replacement therapy, change in serum creatinine and estimated glomerular filtration rate (eGFR), hospital or 30-day mortality, or length of hospital stay. But RIPC significantly increased the minimum eGFR in the IR-AKI subgroup (*P* = 0.006) compared with the control group. In addition, the length of ICU stay in the RIPC group was significantly shorter than in the control group (2.6 vs 2.0 days, *P* = 0.003).

**Conclusions:**

We found strong evidence to support the application of RIPC to prevent CI-AKI, but not IR-AKI.

**Electronic supplementary material:**

The online version of this article (doi:10.1186/s13054-016-1272-y) contains supplementary material, which is available to authorized users.

## Background

Acute kidney injury (AKI) is a common complication in hospitalized patients, especially in the intensive care unit (ICU). Approximately 30–60 % of critically ill patients have AKI [1–3], while the incidence of AKI is about 21.6 % in hospitalized adults [4]. The mortality due to AKI in the ICU can be as high as 60–70 % [5, 6], and in the hospital approximately 20–40 % of patients with AKI die, with mortality being higher in patients with more severe AKI [7, 8]. Although various attempts have been made to prevent or treat AKI, including renoprotective drugs [9] and renal replacement therapy (RRT) [10], most of these efforts have yielded limited success. AKI is still a great burden for patients with risk factors, such as old age, sepsis, hypovolaemia, chronic kidney disease (CKD) and diabetes mellitus.

Remote ischemic preconditioning (RIPC), a technique in which brief episodes of ischemia/reperfusion (IR) applied in distant tissues or organs render the organ resistant to a subsequent sustained episode of ischemia [11], was first proposed [12] and confirmed in the heart [13]. Not only did RIPC have protective effects on the heart, but the concept of RIPC was further extended to reduce the incidence of AKI. RIPC may be a highly appealing, nonpharmacological, practical approach to protect the kidney. Although RIPC’s renoprotection has been demonstrated in animal models [14] of ischemia/reperfusion-induced acute kidney injury (IR-AKI) [15, 16] and contrast-induced acute kidney injury (CI-AKI) [17], its protective effects in clinical settings are still controversial. The authors of one recent meta-analysis [18] concluded that RIPC provides cardiac protection, but there is no evidence of renal protection in patients undergoing cardiac surgery using cardiopulmonary bypass (CPB). Other authors [19] demonstrated that RIPC might be beneficial for the prevention of AKI following cardiac and vascular interventions, but the current evidence is not robust enough to make a recommendation.

In the past year 2015 to 2016, more than ten randomized controlled trials (RCTs) [20–30] were published. These RCTs were not included in previous meta-analyses, and the effects of RIPC on AKI need to be reassessed. However, there may be enough studies to conduct meta-regression analyses to examine associations between effect sizes of RIPC and variables that may influence the efficacy of RIPC, such as comorbidities and surgical procedures. AKI can have a variety of causes, and the effects of RIPC on different cause-specific AKI may also vary. For these reasons, we conducted a systematic review and meta-analysis of RCTs to reassess the effects of RIPC on the incidence and outcomes of AKI and to apply meta-regression analyses of confounders associated with the effects of RIPC on AKI.

## Methods

### Data sources

We performed a computerized search to identify relevant published original studies (1993 to February 2016). The year 1993 was selected as the starting point because it corresponds to the year in which the concept of RIPC was first proposed. The Web of Science, PubMed, Cochrane Library, and OVID databases were searched using medical subject heading terms or keywords. The words searched were “acute kidney injury,” “acute kidney failure,” “acute kidney dysfunction,” “acute kidney insufficiency,” “acute tubular necrosis,” “acute renal failure,” “acute renal injury,” “acute renal dysfunction,” “acute renal insufficiency,” or “contrast induced nephropathy” and “ischemic preconditioning” or “ischemic conditioning.” This search was not limited to the English language or publication type.

### Study selection

An initial eligibility screen of all retrieved titles and abstracts was conducted, and only studies in which researchers reported AKI were selected for further review. The following inclusion criteria were used for final study selection: (1) effects of RIPC on AKI were reported; (2) the protocol was RIPC, not remote ischemic postconditioning or local ischemic conditioning; (3) clear definitions of AKI stated; and (4) at least one of the following outcomes of interest: (a) incidence of AKI, (b) serum creatinine (SCr), or (c) estimated glomerular filtration rate (eGFR) within 72 h after procedures. We restricted the search to clinical RCTs. We excluded studies without clear definitions of AKI or outcomes of interest as well as experimental studies.

### Data extraction and quality assessment

Two reviewers (HJC and LSP) independently examined the studies, and disagreement was resolved by discussion. Data extraction included year of publication, country of origin, study design, sample size, patient characteristics (age and sex), procedures, definitions of AKI, comorbidities, details of RIPC protocols, baseline SCr and eGFR, CPB and cross-clamp time for cardiac surgery, and dose of contrast medium. Our primary endpoint was the incidence of AKI within 72 h after procedures. The secondary endpoints were incidence of AKI stages 1–3, incidence of RRT, changes of SCr and eGFR within 72 h after procedures, hospital or 30-day mortality, length of ICU stay, and length of hospital stay. In this meta-analysis, we categorized the AKI definitions and staging system according to a Kidney Disease: Improving Global Outcomes (KDIGO)-equivalent AKI definition, similarly to previous studies [4, 31]. The study selection, data extraction, and reporting of results were all based on the Preferred Reporting Items for Systematic Reviews and Meta-Analyses checklist [32]. The quality of the studies was assessed independently by pairs of two authors. The Jadad scale (score range 0–5, 5 = best score) was used to quantify the quality of the trials [33].

### Statistical analyses

Comprehensive Meta-Analysis version 2.0 software (Biostat Inc, Englewood, NJ, USA) was used to perform the meta-analysis. Heterogeneity among study point estimates was assessed with the Q-statistic, and the magnitude of heterogeneity being was evaluated with the *I*^2^ index. The random effects model was used for all analyses. Pooled dichotomous data such as incidence of AKI and hospital mortality were expressed as risk ratio (RR) with 95 % CI. Pooled continuous effect measures were expressed as the standardized mean difference with 95 % CI. Publication bias was assessed using funnel plot techniques and the Egger regression test. The random effects meta-regression analyses were performed to evaluate statistically the effects of confounding factors on the renoprotection of RIPC. The variables evaluated by meta-regression were age, percentage of male subjects, percentage of comorbidities, baseline of eGFR, CPB time, cross-clamp time, and dose of contrast medium. All tests of statistical inference reflect a two-sided α of 0.05 or 0.01.

## Results

### Literature search

**Fig. 1:**
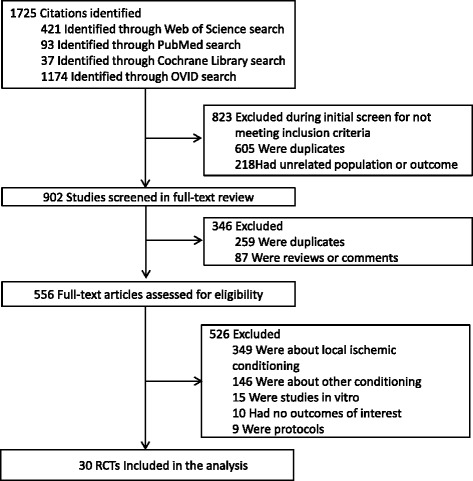
Flow of studies through the review process

In the searches, we identified 1725 records, of which 556 were considered potentially relevant based on title and abstract screening, and we obtained these as full-text studies. There were 30 RCTs including 7244 patients with a median of 96 (interquartile range [IQR] 71–200) patients per study who met our eligibility criteria and were included in this systematic review (Fig. 1). Agreement between investigators at the full-text review stage was excellent, as indicated by a κ of 0.8.

### Study and participant characteristics

Of the included 30 RCTs, one study [34] was available only as an abstract and all others were reported in full-length journal articles. Publication dates ranged from 2007 to 2016 (median 2014). Studies were conducted in a wide range of countries. Twenty trials with an aggregated 6077 patients undergoing cardiac or aortic surgery were assigned to an IR-AKI subgroup, while 10 studies with 1167 patients receiving contrast medium injection were assigned to the CI-AKI subgroup. The proportion of patients who were male ranged from 32.3 % to 92.7 % (median 71.0 %, IQR 57.6–81.2 %), and the patients’ ages ranged from 1 to 76 years (median 66 years, IQR 62–72 years). Additional file 1 shows these details in more detail. Baseline SCr ranged from 33.5 to 143.6 μmol/L (median 95.2 μmol/L, IQR 79.7–102.6 μmol/L), and baseline eGFR ranged from 41.0 to 114.5 ml/min·1.73/m^2^ (median 77.8 ml/min·1.73/m^2^, IQR 51.3–93.2 ml/min·1.73/m^2^). Additional file 2 contains data for the proportions of common comorbidities such as hypertension, diabetes mellitus (DM), dyslipidemia, and previous myocardial infarction; CPB and cross-clamp time; the dose of contrast medium; and definitions of AKI. Overall study quality was good, with a mean Jadad scale score of 3.9 of a possible 5 (median 4, IQR 3–5).

### RIPC protocols

The RIPC methods used varied among studies. An inflatable tourniquet was used around the upper limbs in 22 studies , around the lower limbs in 5 studies, and around both the upper and lower limbs in 1 study [30]. In two studies [35, 36], cross-clamping of the iliac arteries was used. In the majority of studies, cuff pressure was defined as the pressure either up to 200 mmHg or 50 mmHg higher than the systolic arterial pressure. Total ischemic duration ranged from 15 to 30 minutes (median 15, IQR 15–20). Additional file 3 shows these data in more detail.

### Data synthesis

#### Effects of RIPC on the incidence of AKI

**Fig. 2:**
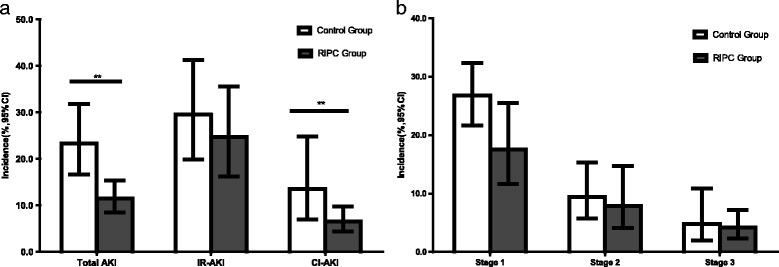
Effects of remote ischemic preconditioning (RIPC) on the incidence of acute kidney injury (AKI). (**a**) Effects of RIPC on total AKI, ischemia/reperfusion-induced AKI (IR-AKI) and contrast-induced AKI (CI-AKI). (**b**) Effects of RIPC on every stage of AKI. **P < 0.01

**Fig. 3:**
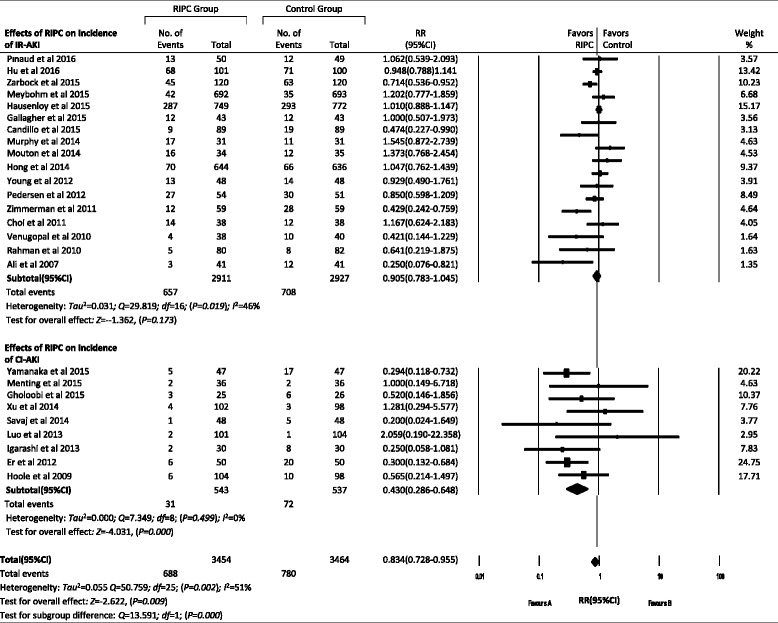
Forest plot showing effects of remote ischemic preconditioning on incidence of acute kidney injury and subgroup analyses. *IR-AKI* ischemia/reperfusion-induced acute kidney injury, *CI-AKI* contrast-induced acute kidney injury, *RIPC* remote ischemic preconditioning, *RR* risk ratio

Data regarding the incidence of AKI were available in 26 trials with an aggregated 7009 patients. The total pooled incidence of AKI in the RIPC group was 11.5 % (95 % CI 8.5–15.3), which was significantly lower than the 23.3 % (95 % CI 16.6–31.8) in the control group (RR 0.834, 95 % CI 0.728–0.955, *P* = 0.009). Nine studies with an aggregated 2504 patients after cardiac surgery provided the incidence in every stage of AKI. The pooled incidence rates were 17.5 % (95 % CI 11.6–25.5), 7.9 % (95 % CI 4.1–14.7), and 4.2 % (95 % CI 2.3–7.2) for stage 1, stage 2, and stage 3 AKI, respectively, in the RIPC group. The corresponding rates were 26.8 % (95 % CI 21.7–32.4), 9.4 % (95 % CI 5.7–15.3), and 4.8 % (95 % CI 2.0–10.9) in the control group. But there were no significant differences in every stage of AKI. Contradictorily, the incidence of RRT was slightly higher in the RIPC group than in the control group (4.6 % [95 % CI 2.7–7.8] vs 3.2 % [95 % CI 1.6–6.4], RR 1.116 [95 % CI 0.524–2.377], *P* = 0.776). In addition, RIPC significantly reduced the incidence of AKI in the CI-AKI subgroup from 13.5 % to 6.5 % (RR 0.430, 95 % CI 0.286–0.648, *P* = 0.000), but not in the IR-AKI subgroup, in which it reduced the incidence from 29.5 % to 24.7 % (RR 0.905, 95 % CI 0.783–1.045, *P* = 0.173). There was a significant difference between these two subgroups (*P* = 0.001) (Figs. 2 and 3).

#### Meta-regression analyses

**Fig. 4:**
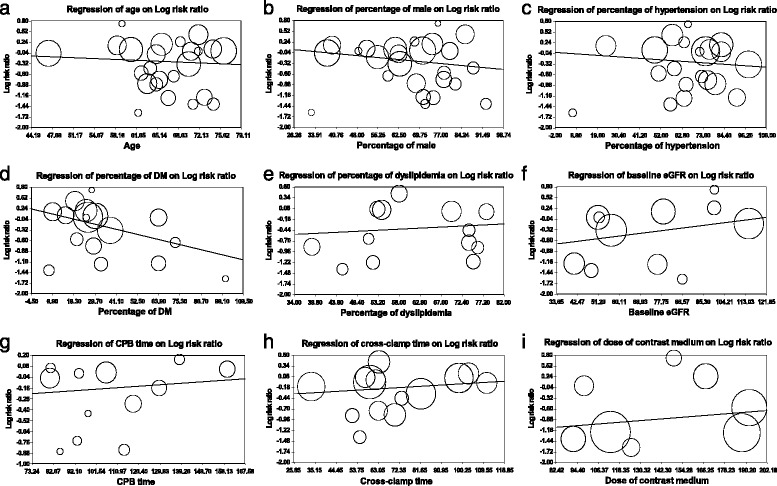
Meta-regression results of reduction of acute kidney injury (AKI) by remote ischemic preconditioning (RIPC). Meta-regression of age (**a**), percentage of male (**b**), percentage of hypertension (**c**), percentage of diabetes mellitus (DM) (**d**), percentage of dyslipidemia (**e**), baseline estimated glomerular filtration (eGFR) (**f**), cardiopulmonary bypass (CPB) time (**g**), cross-clamp time (**h**) and dose of contrast medium (**i**) on log risk ratios

Random effects meta-regression showed that RIPC tended to strengthen its renoprotection with a significant difference (*q* = 3.95, *df* = 1, *P* = 0.047) along with a higher percentage of DM. We did not find any other significant correlation between the incidence of AKI and probable confounding factors such as age, percentage of male patients, other comorbidities, baseline eGFR, CPB and cross-clamp time, and dose of contrast medium (Fig. 4).

#### Effects of RIPC on SCr and eGFR

**Fig. 5:**
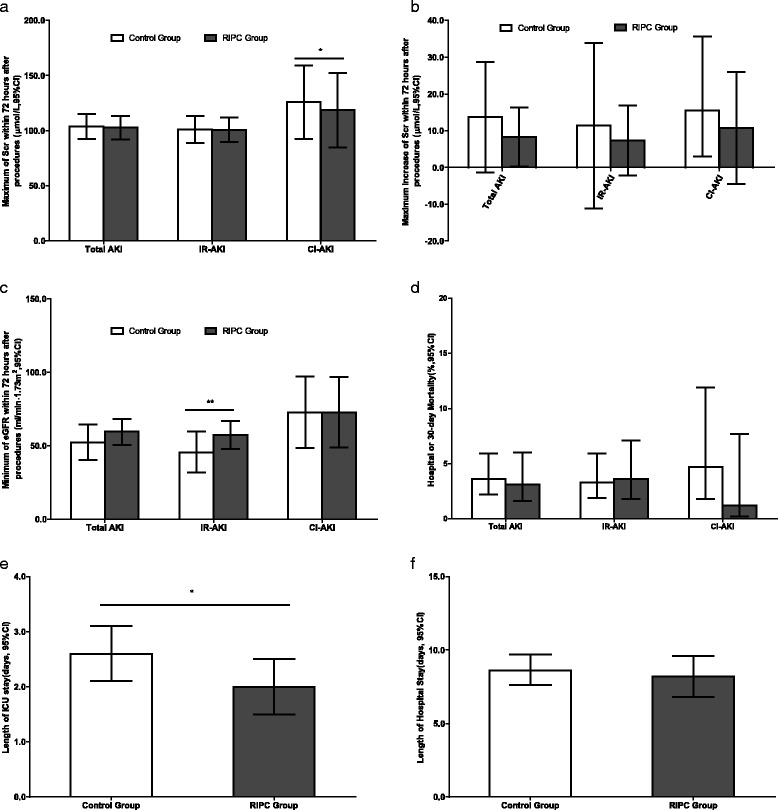
Effects of remote ischemic preconditioning (RIPC) on outcomes of acute kidney injury (AKI). Effects of RIPC on maximum of serum creatinine (Scr) (**a**), maximum increase of Scr (**b**) and minimum of estimated glomerular filtration (eGFR) (**c**) within 72 hours after procedures, hospital or 30-day mortality (**d**), length of intensive care unit (ICU) (**e**) and hospital stay (**f**). IR-AKI ischemia/reperfusion-induced acute kidney injury, CI-AKI contrast-induced acute kidney injury. *P < 0.05, **P < 0.01

**Fig. 6:**
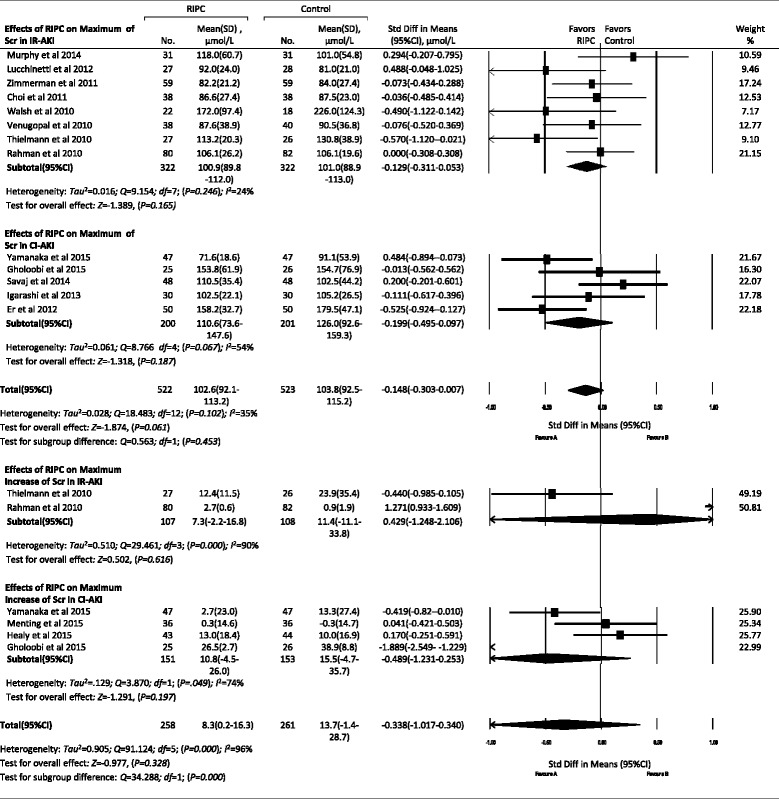
Forest plot showing effects of remote ischemic preconditioning on the change of serum creatinine within 72 h after procedures. *IR-AKI* ischemia/reperfusion-induced acute kidney injury, *CI-AKI* contrast-induced acute kidney injury, *RIPC* remote ischemic preconditioning, *SD* standard deviation, *Std Diff* standard difference

**Fig. 7:**
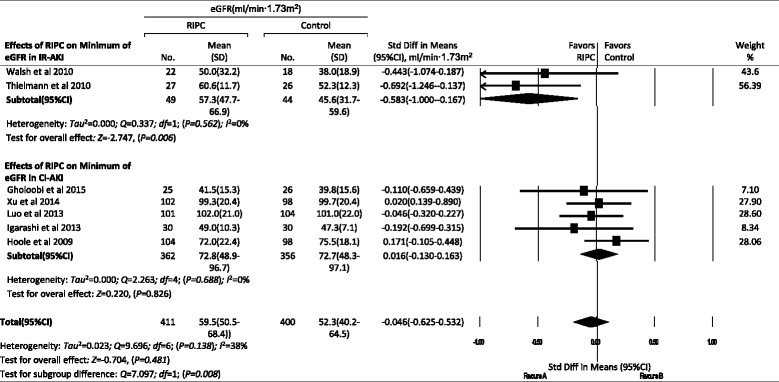
Forest plot showing effects of remote ischemic preconditioning on the change of estimated glomerular filtration rate within 72 h after procedures. *IR-AKI* ischemia/reperfusion-induced acute kidney injury, *CI-AKI* contrast-induced acute kidney injury, *RIPC* remote ischemic preconditioning, *SD* standard deviation, *Std Diff* standard difference

Data about maximum SCr values within 72 h after procedures were available in 13 trials; maximum increase of SCr was available in 6 trials; and minimum eGFR values were available in 7 trials. There were no significant differences in these indexes for total AKI and subgroups between the control and RIPC groups, except that RIPC increased the minimum eGFR in the IR-AKI subgroup (*P* = 0.006) compared with the control group (Figs. 5, 6 and 7).

#### Effects of RIPC on other AKI outcomes

**Fig. 8:**
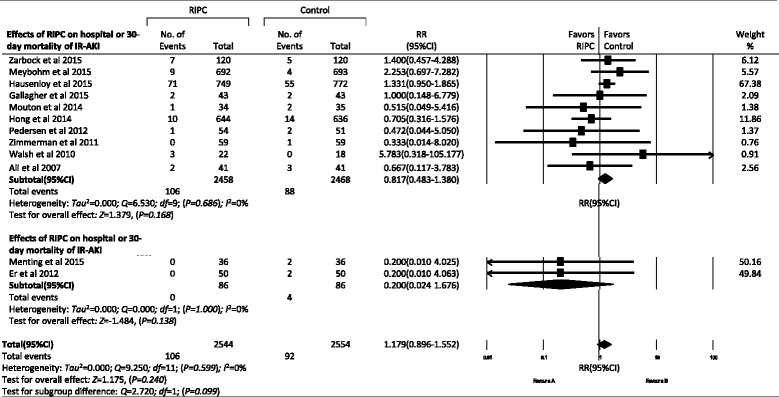
Forest plot showing effects of remote ischemic preconditioning on hospital or 30-day mortality due to acute kidney injury. *IR-AKI* ischemia/reperfusion-induced acute kidney injury, *CI-AKI* contrast-induced acute kidney injury, *RIPC* remote ischemic preconditioning, *RR* risk ratio

**Fig. 9:**
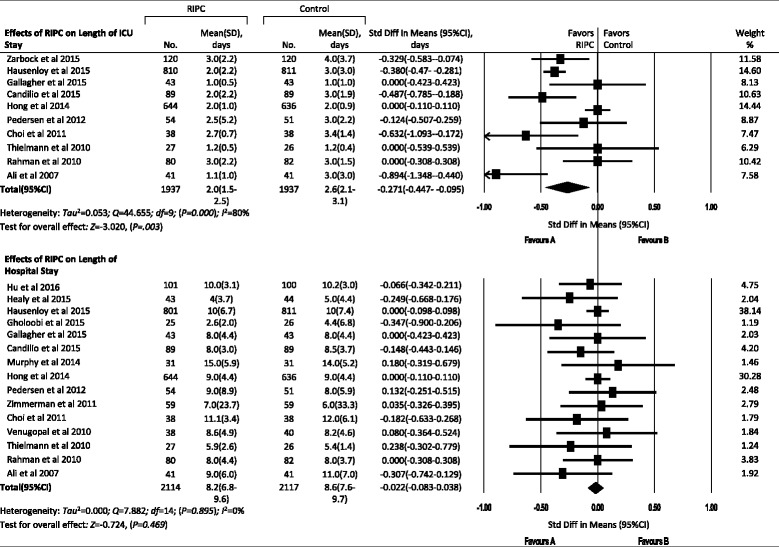
Effects of remote ischemic preconditioning on length of intensive care unit and hospital length of stay for acute kidney injury. *ICU* intensive care unit, *RIPC* remote ischemic preconditioning, *SD* standard deviation, *Std Diff* standard difference

Hospital or 30-day mortality rates were reported in 12 trials with an aggregated 5098 patients, but no significant differences were observed between the RIPC and control groups in total AKI (3.1 % vs 3.6 %, RR, 1.179, 95 % CI, 0.896–1.552, *P* = 0.240) and subgroup analyses (Figs. 5 and 8). In 10 trials with an aggregated 3874 patients, researchers reported the length of ICU stay, and in 15 trials with an aggregated 4231 patients investigators reported the length of hospital stay. RIPC reduced the length of ICU stay from 2.6 days (95 % CI 2.1–3.1) to 2.0 days (95 % CI 1.5–2.5) (standard difference in means −0.271, 95 % CI −0.447 to −0.095, *P* = 0.003), but it did not reduce the length of hospital stay (standard difference in means −0.022, 95 % CI −0.083 to 0.038, *P* = 0.469) (Figs. 5 and 9).

#### Publication bias

**Fig. 10:**
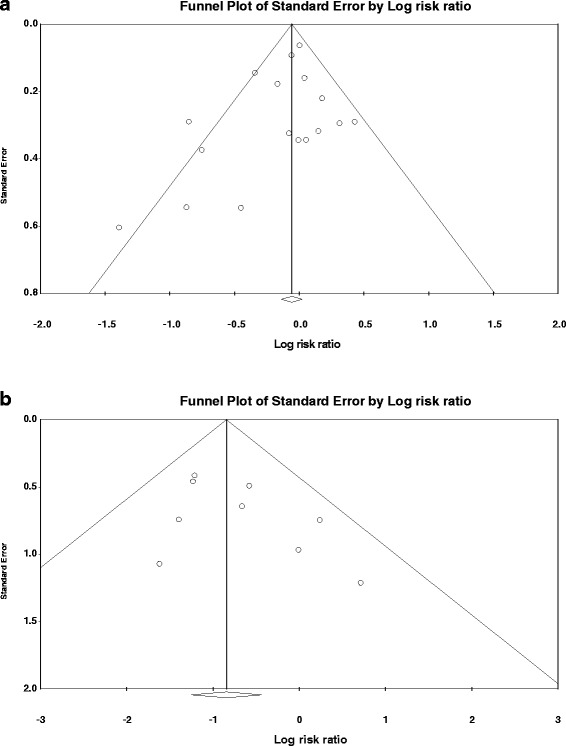
Funnel plot to evaluate for publication bias for effects of remote ischemic preconditioning on incidence of acute kidney injury

The funnel plots showed no evidence of publication bias. Egger’s test for a regression intercept gave *P* values of 0.177 for effects of RIPC on incidence of IR-AKI (Fig. 10a) and 0.178 for effects of RIPC on incidence of CI-AKI (Fig. 10b).

## Discussion

We conducted an extensive, systematic review of the protective effects of RIPC on AKI in the clinical setting. With data from 30 RCTs comprising a total of 7244 patients, this analysis included substantially more trials than previous published meta-analyses addressing this question. It also provides a comparison of the protection of RIPC on different cause-specific AKIs. In meta-regression analyses, we tried to find the patients who were most likely to benefit from RIPC.

### Effects of RIPC on the incidence of total AKI

The main finding of this meta-analysis was that RIPC significantly decreased the incidence of AKI from 23.3 % to 11.5 % (RR, 0.834, 95 % CI, 0.728–0.955, *P* = 0.009). To our knowledge, this is the first meta-analysis to come to this conclusion. In 2014, Yang et al [19] conducted a meta-analysis with 13 trials and 1334 patients and concluded that, compared with the control group, RIPC decreased the risk of AKI for patients undergoing cardiac and vascular interventions with marginal statistical significance (*P* = 0.06). Similarly, Li et al [37] concluded that there was not a lower incidence of AKI in patients undergoing cardiac and vascular interventions in the RIPC group than in the control group (*P* = 0.10). In our meta-analysis, data regarding the incidence of AKI were available in 26 trials comprising 7009 patients; half of these trials were newly published during 2014–2015 and were not included in previous meta-analyses. Statistically, RIPC could lead to a 17 % decrease in the risk of AKI.

### Different effects of RIPC on the incidence of CI-AKI and IR-AKI

In further subgroup analysis, we found that RIPC significantly reduced the incidence of AKI in the CI-AKI subgroup (*P* = 0.000), but not in the IR-AKI subgroup (*P* = 0.173). IR was the main mechanism of AKI after cardiac surgery, while the use of contrast medium led mainly to renal injury in percutaneous coronary intervention (PCI) or contrast-enhanced computed tomography. Similarly, Yasin et al [18] did not find renoprotection of RIPC after cardiac surgery (*P* = 0.07), while Pei et al [38] concluded that RIPC significantly reduced the perioperative incidence of CI-AKI in patients undergoing elective coronary intervention (*P* = 0.04). We also analyzed the effect size of RIPC on IR-AKI and CI-AKI, and we found a significant subgroup difference (*P* = 0.000). So, patients who are at risk of CI-AK might benefit more than those at risk of IR-AKI.

### Confounding factors that influenced the effects of RIPC

Various confounding factors influenced the effects of RIPC on AKI. We found that a higher percentage of patients with DM gained more benefits, with statistical significance (*P* = 0.047), when random effects regression was used, which was opposite to our previous understanding. One trial [39] indicated that RIPC significantly reduces the incidence of contrast-induced nephrology in patients without diabetes, but not in those with diabetes, undergoing PCI. Schenning et al [40] also reported that ischemic preconditioning protected healthy but not hyperglycemic glomerular endothelial monolayers from IR injury. Wouter et al [41] concluded that DM does not abolish, but might reduce, the cardioprotective effect of ischemic postconditioning. In addition, age, sex, other comorbidities, CPB and cross-clamp time, and dose of contrast medium may also be confounders of RIPC, but there were no significant differences. In all, the effects of RIPC on patients with a high risk of AKI need to be reassessed in the future.

### Effects of RIPC on other outcomes of AKI

In this meta-analysis, we did not find the effects of RIPC on the incidence of AKI stages 1–3 or the incidence of RRT. RIPC also did not reduce the hospital or 30-day mortality or the length of hospital stay. Although there were no significant differences in SCr and eGFR in total AKI between the control and RIPC groups, RIPC increased the minimum eGFR in the IR-AKI subgroup (*P* = 0.006) and reduced the length of ICU stay from 2.6 to 2.0 days (*P* = 0.003), which was not reported in previous meta-analyses.

### Study limitations

It is important to note the limitations of our study. First, the RIPC protocol should have an important influence on its effects on AKI; however, with the limited data and high heterogeneity in our present analysis, we cannot conclude which protocol was superior to another (for example, early or late RIPC, RIPC on arms or legs, and so forth). Second, many confounding factors impact the effects of RIPC, and meta-regression may not be enough to verify this issue. Further clinical studies are needed to test the renoprotection of RIPC in patients with high-risk conditions. Third, it may be improper to define AKI after cardiac surgery to be IR-AKI and to define AKI after contrast medium injection to be CI-AKI, because there were many other risk factors that could have caused AKI in these situations.

## Conclusions

We found strong evidence to support the application of RIPC for prevention of CI-AKI but not IR-AKI. We found low-quality evidence suggesting that RIPC was associated with improvements in hospital mortality and hospital length of stay. The various effects of RIPC on AKI at different levels of risk need to be tested in future.

## Additional files

Additional file 1: Demographic data of included clinical trials. *AAA* abdominal aortic aneurysm, *CABG* coronary artery bypass grafting, *CECT* contrast-enhanced computed tomography, *PCI* percutaneous coronary intervention. (DOCX 63 kb) Additional file 2: Details of included clinical trials. *DM* diabetes mellitus, *MI* myocardial infarction, *KDIGO* Kidney Disease: Improving Global Outcomes, *NR* not reported, *AKIN* Acute Kidney Injury Network, *L-FABP* liver-type fatty acid-binding protein, *RIFLE* risk, injury, failure, loss, end-stage renal disease. (DOCX 65 kb) Additional file 3: Details of remote ischemic preconditioning in clinical trials. *IR* ischemia reperfusion. (DOCX 59 kb)

## Abbreviations

AAA: abdominal aortic aneurysm
AKI: acute kidney injury
AKIN: Acute Kidney Injury Network
CABG: coronary artery bypass grafting
CECT: contrast-enhanced computed tomography
CI-AKI: contrast-induced acute kidney injury
CPB: cardiopulmonary bypass
DM: diabetes mellitus
eGFR: estimated glomerular filtration rate
ICU: intensive care unit
IQR: interquartile range
IR: ischemia/reperfusion
IR-AKI: ischemia/reperfusion-induced acute kidney injury
KDIGO: Kidney Disease: Improving Global Outcomes
L-FABP: liver-type fatty acid-binding protein
MI: myocardial infarction
NR: not reported
PCI: percutaneous coronary intervention
RCT: randomized controlled trial
RIFLE: risk, injury, failure, loss, end-stage renal disease
RIPC: remote ischemic preconditioning
RR: risk ratio
RRT: renal replacement therapy
SCr: serum creatinine
SD: standard deviation
Std Diff: standard difference
