# Supplementary material for: Protection of remote ischemic preconditioning against acute kidney injury: a systematic review and meta-analysis
Source: Crit Care. 2016 Apr 20;20:111. doi: 10.1186/s13054-016-1272-y (PMC4837562; doi:10.1186/s13054-016-1272-y)
Supplement: Additional file 1: — Demographic data of included clinical trials. AAA abdominal aortic aneurysm, CABG coronary artery bypass grafting, CECT contrast-enhanced computed tomography, PCI percutaneous coronary intervention. (DOCX 63 kb) [file 13054_2016_1272_MOESM1_ESM.docx]

Additional file 1. Demographic data of included clinical trials

| Source | Country | Study Design | Procedure | No. of Patients | Male (%) | Mean Age(Y) | | No. of Patients | | Jadad scale |
| --- | --- | --- | --- | --- | --- | --- | --- | --- | --- | --- |
|  |  |  |  |  |  | Control | RIPC | Control | RIPC |  |
| Pinaud et al[1] 2016 | France | Prospective, randomized, single-blinded (patients) controlled trial with blinded-endpoint analysis | Elective aortic valve replacement without combined surgery with CABG | 99 | 51(51.5) | 72.9 | 75.8 | 49 | 50 | 5 |
| Hu et al[(] 2016 | China | Single-center, randomized, prospective, and double-blinded study | Valve replacement surgery | 201 | 76(37.8) | 46.7 | 47.4 | 100 | 101 | 5 |
| Zarbock et al[[3](#_ENREF_3)] 2015 | Germany | Multicenter, randomized, controlled, clinical trial | Cardiac surgery | 240 | 151(62.9) | 70.6 | 70.1 | 120 | 120 | 3 |
| Yamanaka et al[[4](#_ENREF_4)] 2015 | Japan | Prospective, single-blind, multicenter, randomized, sham-controlled parallel-group study | PCI | 94 | 70(74.5) | 67 | 67 | 47 | 47 | 5 |
| Meybohm et al[[5](#_ENREF_5)] 2015 | Germany | Prospective, randomized, double-blind,multicenter, parallel-group, controlled trial | Elective cardiovascular surgery | 1385 | 1028(74.2) | 66.0 | 65.8 | 693 | 692 | 5 |
| Menting et al[[6](#_ENREF_6)] 2015 | The Netherlands | Multicenter, single blinded, randomized controlled trial | Interventional or diagnostic radiological procedure | 72 | 35(48.6) | 73 | 71 | 36 | 36 | 5 |
| Healy et al[[7](#_ENREF_7)] 2015 | Ireland | Randomized trial | CECT scan | 87 | 49(56.3) | 62 | 63 | 44 | 43 | 2 |
| Hausenloy et al[[8](#_ENREF_8)] 2015 | UK | Multicenter, randomized, sham-controlled clinical study | On-pump CABG | 1612 | 1142(70.8) | 76.3 | 76.1 | 811 | 801 | 3 |
| Gholoobi et al[[9](#_ENREF_9)] 2015 | Iran | Double-blind randomized clinical trial | Coronary angiography and/or angioplasty | 51 | 30(58.8) | 67.1 | 68.7 | 26 | 25 | 5 |
| Gallagher et al[[10](#_ENREF_10)] 2015 | UK | Prospective and randomized controlled trial | CABG | 86 | 69(80.2) | 72.8 | 68.7 | 43 | 43 | 3 |
| Candilio et al[[11](#_ENREF_11)] 2015 | UK | Double-blinded randomized controlled clinical trial | Cardiac surgery | 178 | 139(78.1) | 66 | 65 | 89 | 89 | 5 |
| Xu et al[[12](#_ENREF_12)] 2014 | China | Single-center, randomized, and prospective study | PCI | 200 | 136(68.0) | 68.9 | 69.1 | 98 | 102 | 3 |
| Savaj et al[[13](#_ENREF_13)] 2014 | Iran | Randomized control trial | Coronary angiography | 96 | 31(32.3) | 60.9 | 63.0 | 48 | 48 | 2 |
| Murphy et al[[14](#_ENREF_14)] 2014 | UK | Prospective, randomized, double-blind, single-center study | Elective AAA repair | 62 | 53(85.5) | 69 | 75 | 31 | 31 | 5 |
| Mouton et al[[15](#_ENREF_15)] 2014 | Italy | Randomized controlled trial | Elective AAA repair | 69 | NR | NR | NR | 35 | 34 | 2 |
| Hong et al[[16](#_ENREF_16)] 2014 | South Korea | Prospective randomized controlled study | Cardiac surgery | 1280 | 785(61.3) | 60.9 | 60.8 | 636 | 644 | 3 |
| Luo et al[[17](#_ENREF_17)] 2013 | China | Single-center, randomized controlled trial | PCI | 205 | 156(76.1) | 59.3 | 59.2 | 104 | 101 | 3 |
| Igarashi et al[[18](#_ENREF_18)] 2013 | Japan | Prospective, randomized, non-blinded, multicenter study | Coronary angiography | 60 | 43(71.7) | 70.8 | 71.3 | 30 | 30 | 3 |
| Young et al[[19](#_ENREF_19)] 2012 | New Zealand | Double blind, parallel-groups, phase 2b randomized controlled trial | Cardiac surgery | 96 | 60(62.5) | 64.4 | 65.5 | 48 | 48 | 5 |
| Pedersen et al[[20](#_ENREF_20)] 2012 | Denmark | Single center, stratified, double-blind, parallel-group, randomized controlled trial | Cardiac surgery | 105 | 58(55.2) | 0.9 | 1.0 | 51 | 54 | 5 |
| Lucchinetti et al[[21](#_ENREF_21)] 2012 | Canada | Randomized placebo-controlled trial | Cardiac surgery | 55 | 50(90.9) | 62 | 59 | 28 | 27 | 3 |
| Er et al[[22](#_ENREF_22)] 2012 | Germany | Prospective, randomized, blinded, sham-controlled, single center pilot trial | Coronary angiography | 100 | 71(71.0) | 72.7 | 73.2 | 50 | 50 | 5 |
| Zimmerman et al[[23](#_ENREF_23)] 2011 | USA | Randomized, single-blind, and controlled pilot trial | Cardiac surgery | 118 | 81(68.6) | 65 | 62 | 59 | 59 | 5 |
| Choi et al[[24](#_ENREF_24)] 2011 | Republic of Korea | Prospective and randomized controlled trial | Complex valvular heart surgery | 76 | 30(39.5) | 60 | 57 | 38 | 38 | 3 |
| Walsh et al[[25](#_ENREF_25)]  2010 | UK | Randomized controlled trial | Elective AAA repair | 40 | 34(85.0) | 72 | 75 | 18 | 22 | 2 |
| Venugopal et al[[26](#_ENREF_26)] 2010 | UK | Single-blind randomized controlled study | CABG | 78 | 64(82.1) | 66 | 64 | 40 | 38 | 5 |
| Thielmann et al[[27](#_ENREF_27)] 2010 | Germany | Prospective, single-center, single-blinded, randomized protocol | CABG | 53 | 45(84.9) | 64.1 | 63.4 | 26 | 27 | 5 |
| Rahman et al[[28](#_ENREF_28)] 2010 | UK | Single-center, prospective, randomized, placebo-controlled trial | CABG | 162 | 143(88.3) | 65 | 63 | 82 | 80 | 3 |
| Hoole et al[[29](#_ENREF_29)] 2009 | UK | Prospective, single-center, randomized controlled trial | PCI | 202 | 158(78.2) | 61.8 | 63.2 | 98 | 104 | 3 |
| Ali et al[[30](#_ENREF_30)] 2007 | UK | Randomized, double-blind, controlled trial | Elective AAA Repair | 82 | 76(92.7) | 75 | 74 | 41 | 41 | 5 |

Abbreviations: UK, United Kingdom; USA, United States of America; PCI, percutaneous coronary intervention; CECT, contrast-enhanced computed tomography; CABG, coronary artery bypass grafting; AAA, abdominal aortic aneurysm.

**References**

1. Pinaud F, Corbeau J-J, Baufreton C, Binuani J-P, De Brux J-L, Fouquet O, Angoulvant D, Furber A, Prunier F: **Remote ischemic preconditioning in aortic valve surgery: Results of a randomized controlled study**. *J Cardiol* 2016, **67**:36-41.

2. Hu Q, Luo W, Huang L, Huang R, Chen R, Gao Y: **Multiorgan protection of remote ischemic perconditioning in valve replacement surgery**. *J Surg Res* 2016, **200**:13-20.

3. Zarbock A, Schmidt C, Van Aken H, Wempe C, Martens S, Zahn PK, Wolf B, Goebel U, Schwer CI, Rosenberger P *et al*: **Effect of Remote Ischemic Preconditioning on Kidney Injury Among High-Risk Patients Undergoing Cardiac Surgery A Randomized Clinical Trial**. *J Am Med Assoc* 2015, **313**:2133-2141.

4. Yamanaka T, Kawai Y, Miyoshi T, Mima T, Takagaki K, Tsukuda S, Kazatani Y, Nakamura K, Ito H: **Remote ischemic preconditioning reduces contrast-induced acute kidney injury in patients with ST-elevation myocardial infarction: A randomized controlled trial**. *Int J Cardiol* 2015, **178**:136-141.

5. Meybohm P, Bein B, Brosteanu O, Cremer J, Gruenewald M, Stoppe C, Coburn M, Schaelte G, Boening A, Niemann B *et al*: **A Multicenter Trial of Remote Ischemic Preconditioning for Heart Surgery**. *New Engl J Med* 2015, **373**:1397-1407.

6. Menting TP, Sterenborg TB, de Waal Y, Donders R, Wever KE, Lemson MS, van der Vliet JA, Wetzels JF, SchultzeKool LJ, Warle MC: **Remote Ischemic Preconditioning To Reduce Contrast-Induced Nephropathy: A Randomized Controlled Trial**. *Eur J Vasc Endovasc* 2015, **50**:527-532.

7. Healy DA, Feeley I, Keogh CJ, Scanlon TG, Hodnett PA, Stack AG, Moloney MC, Whittaker P, Walsh SR: **Remote ischemic conditioning and renal function after contrast-enhanced CT scan: A randomized trial**. *Clinl Invest Med Medicine* 2015, **38**:E110-E118.

8. Hausenloy DJ, Candilio L, Evans R, Ariti C, Jenkins DP, Kolvekar S, Knight R, Kunst G, Laing C, Nicholas J *et al*: **Remote Ischemic Preconditioning and Outcomes of Cardiac Surgery**. *New Engl J Med* 2015, **373**:1408-1417.

9. Gholoobi A, Sajjadi SM, Shabestari MM, Eshraghi A, Shamloo AS: **The Impact of Remote Ischemic Pre-Conditioning on Contrast-Induced Nephropathy in Patients Undergoing Coronary Angiography and Angioplasty: A Double-Blind Randomized Clinical Trial**. *Electron physician* 2015, **7**:1557-1565.

10. Gallagher SM, Jones DA, Kapur A, Wragg A, Harwood SM, Mathur R, Archbold RA, Uppal R, Yaqoob MM: **Remote ischemic preconditioning has a neutral effect on the incidence of kidney injury after coronary artery bypass graft surgery.** *Kidney Int* 2015, **87**: 473-481.

11. Candilio L, Malik A, Ariti C, Barnard M, Salvo C, Lawrence D, Hayward M, Yap J, Roberts N, Sheikh A *et al*: **Effect of remote ischaemic preconditioning on clinical outcomes in patients undergoing cardiac bypass surgery: a randomised controlled clinical trial**. *Heart* 2015, **101**: 185-192.

12. Xu XH, Zhou YJ, Luo SJ, Zhang WJ, Zhao YX, Yu M, Ma Q, Gao F, Shen H, Zhang JW: **Effect of Remote Ischemic Preconditioning in the Elderly Patients With Coronary Artery Disease With Diabetes Mellitus Undergoing Elective Drug-Eluting Stent Implantation**. *Angiology* 2014, **65**:660-666.

13. Savaj S, Savoj J, Jebraili I, Sezavar SH: **Remote ischemic preconditioning for prevention of contrast-induced acute kidney injury in diabetic patients**. IranJ Kidney Dis 2014, 8: 457-460.

14. Murphy N, Vijayan A, Frohlich S, O'Farrell F, Barry M, Sheehan S, Boylan J, Conlon N: **Remote ischemic preconditioning does not affect the incidence of acute kidney injury after elective abdominal aortic aneurysm repair**. *J Cardiothorac Vasc Anesth* 2014,**28**: 1285-1292.

15. Mouton R, Pollock J, Soar J, Mitchell D, Rogers C: **Remote ischaemic preconditioning for elective abdominal aortic aneurysm (AAA) repair: a randomized controlled trial to assess feasibility**. *Appl Cardiopulm Pathophysiol* 2014, **18**: 35.

16. Hong DM, Lee E-H, Kim HJ, Min JJ, Chin J-H, Choi D-K, Bahk J-H, Sim J-Y, Choi I-C, Jeon Y: **Does remote ischaemic preconditioning with postconditioning improve clinical outcomes of patients undergoing cardiac surgery? Remote Ischaemic Preconditioning with Postconditioning Outcome Trial**. *Eur Heart J* 2014, **35**:176-183.

17. Luo SJ, Zhou YJ, Shi DM, Ge HL, Wang JL, Liu RF: **Remote Ischemic Preconditioning Reduces Myocardial Injury in Patients Undergoing Coronary Stent Implantation**. *Can J Cardiol* 2013, **29**:1084-1089.

18. Igarashi G, Iino K, Watanabe H, Ito H: **Remote Ischemic Pre-Conditioning Alleviates Contrast-Induced Acute Kidney Injury in Patients With Moderate Chronic Kidney Disease**. *Circ J* 2013, **77**:3037-3044.

19. Young PJ, Dalley P, Garden A, Horrocks C, La Flamme A, Mahon B, Miller J, Pilcher J, Weatherall M, Williams J *et al*: **A pilot study investigating the effects of remote ischemic preconditioning in high-risk cardiac surgery using a randomised controlled double-blind protocol**. *Basic Res Cardiol* 2012, **107**:256.

20. Pedersen KR, Ravn HB, Povlsen JV, Schmidt MR, Erlandsen EJ, Hjortdal VE: **Failure of remote ischemic preconditioning to reduce the risk of postoperative acute kidney injury in children undergoing operation for complex congenital heart disease: A randomized single-center study**. *J Thorac Cardiov Sur* 2012, **143**:576-583.

21. Lucchinetti E, Bestmann L, Feng JH, Freidank H, Clanachan AS, Finegan BA, Zaugg M: **Remote Ischemic Preconditioning Applied during Isoflurane Inhalation Provides No Benefit to the Myocardium of Patients Undergoing On-pump Coronary Artery Bypass Graft Surgery Lack of Synergy or Evidence of Antagonism in Cardioprotection?** *Anesthesiology* 2012, **116**:296-310.

22. Er F, Nia AM, Dopp H, Hellmich M, Dahlem KM, Caglayan E, Kubacki T, Benzing T, Erdmann E, Burst V *et al*: **Ischemic Preconditioning for Prevention of Contrast Medium-Induced Nephropathy Randomized Pilot RenPro Trial (Renal Protection Trial)**. *Circulation* 2012, **126**:296-303.

23. Zimmerman RF, Ezeanuna PU, Kane JC, Cleland CD, Kempananjappa TJ, Lucas FL, Kramer RS: **Ischemic preconditioning at a remote site prevents acute kidney injury in patients following cardiac surgery**. *Kidney Int* 2011, **80**:861-867.

24. Choi YS, Shim JK, Kim JC, Kang KS, Seo YH, Ahn KR, Kwak YL: **Effect of remote ischemic preconditioning on renal dysfunction after complex valvular heart surgery: a randomized controlled trial**. *J Thorac Cardiov Sur* 2011, **142:** 148-154.

25. Walsh SR, Sadat U, Boyle JR, Tang TY, Lapsley M, Norden AG, Gaunt ME: **Remote Ischemic Preconditioning for Renal Protection During Elective Open Infrarenal Abdominal Aortic Aneurysm Repair: Randomized Controlled Trial**. *Vasc Endovasc Surg* 2010, **44**:334-340.

26. Venugopal V, Laing CM, Ludman A, Yellon DM, Hausenloy D: **Effect of Remote Ischemic Preconditioning on Acute Kidney Injury in Nondiabetic Patients Undergoing Coronary Artery Bypass Graft Surgery: A Secondary Analysis of 2 Small Randomized Trials**. *Am J Kidney Dis* 2010, **56**:1043-1049.

27. Thielmann M, Kottenberg E, Boengler K, Raffelsieper C, Neuhaeuser M, Peters J, Jakob H, Heusch G: **Remote ischemic preconditioning reduces myocardial injury after coronary artery bypass surgery with crystalloid cardioplegic arrest**. *Basic Res Cardiol* 2010, **105**:657-664.

28. Rahman IA, Mascaro JG, Steeds RP, Frenneaux MP, Nightingale P, Gosling P, Townsend P, Townend JN, Green D, Bonser RS: **Remote ischemic preconditioning in human coronary artery bypass surgery: from promise to disappointment?** *Circulation* 2010, **122**: S53-59

29. Hoole SP, Heck PM, Sharples L, Khan SN, Duehmke R, Densem CG, Clarke SC, Shapiro LM, Schofield PM, O'Sullivan M *et al*: **Cardiac Remote Ischemic Preconditioning in Coronary Stenting (CRISP Stent) Study A Prospective, Randomized Control Trial**. *Circulation* 2009, **119**:820-827.

30. Ali ZA, Callaghan CJ, Lim E, Ali AA, Nouraei SAR, Akthar AM, Boyle JR, Varty K, Kharbanda RK, Dutka DP *et al*: **Remote ischemic preconditioning reduces myocardial and renal injury after elective abdominal aortic aneurysm repair - A randomized controlled trial**. *Circulation* 2007, **116**:I98-I105.
