# Supplementary material for: Protection of remote ischemic preconditioning against acute kidney injury: a systematic review and meta-analysis
Source: Crit Care. 2016 Apr 20;20:111. doi: 10.1186/s13054-016-1272-y (PMC4837562; doi:10.1186/s13054-016-1272-y)
Supplement: Additional file 2: — Details of included clinical trials. DM diabetes mellitus, MI myocardial infarction, KDIGO Kidney Disease: Improving Global Outcomes, NR not reported, AKIN Acute Kidney Injury Network, L-FABP liver-type fatty acid-binding protein, RIFLE risk, injury, failure, loss, end-stage renal disease. (DOCX 65 kb) [file 13054_2016_1272_MOESM2_ESM.docx]

Additional file 2.Details of included clinical trials

| Source | Comorbidities (%) | | | | Baseline Scr  (μmol/L) | | Baseline eGFR  (ml/min·1.73m2) | | CPB time  (min) | | Cross-clamp time (min) | | Contrast medium  (ml) | | Definitions of AKI |
| --- | --- | --- | --- | --- | --- | --- | --- | --- | --- | --- | --- | --- | --- | --- | --- |
|  | Hypertension | DM | Dyslipidemia | Previous MI | Control | RIPC | Control | RIPC | Control | RIPC | Control | RIPC | Control | RIPC |  |
| Pinaud et al[[1](#_ENREF_1)] 2016 | 77(77) | 14(14) | 53(530 | NR | NR | NR | NR | NR | 79.9±21.8 | 82.9±30.2 | 55.3±18.8 | 59.5±25.2 | — | — | AKIN |
| Hu et al[[2](#_ENREF_2)] 2016 | NR | NR | NR | NR | NR | NR | NR | NR | 84.3±36.7 | 77.9±29.2 | 61.3±30.2 | 57.5±25.3 | — | — | AKIN |
| Zarbock et al[[3](#_ENREF_3)] 2015 | 232  (97) | 90  (38) | NR | NR | 106.1±35.4 | 97.2±35.4 | 56.4±15.8 | 56.7±13.4 | 116±55.9 | 120.0±37.4 | 78.0±39.6 | 86±29.7 | — | — | KDIGO |
| Yamanaka et al[[4](#_ENREF_4)] 2015 | 60  (64) | 31  (33) | 49  (52) | 52  (55) | 76.9±38.9 | 72.5±18.6 | 79±33 | 73±20 | — | — | — | — | 199±87 | 177±53 | Scr↑>0.5 mg/dL or 25% from the baseline value |
| Meybohm et al[[5](#_ENREF_5)] 2015 | 1146(83) | 344(25) | NR | 400(29) | NR | NR | NR | NR | NR | NR | NR | NR | — | — | Acute renal failure |
| Menting et al[[6](#_ENREF_6)] 2015 | 52(72) | 18(25) | NR | 29(40) | 119.0±32.0 | 115.0±70.0 | 52.0±13.0 | 51.0±11.0 | — | — | — | — | 98±29 | 99±29 | Scr↑>0.5 mg/dL or 25% from the baseline value |
| Healy et al[[7](#_ENREF_7)] 2015 | 37  (43) | 13  (15) | NR | NR | 75±17.0 | 73±19.3 | NR | NR | — | — | — | — | 90 | 90 | NR |
| Hausenloy et al[[8](#_ENREF_8)] 2015 | 1201(75) | 414(26) | 1125(70) | 637(40) | NR | NR | NR | NR | 107±291 | 105±408 | 71±205 | 69±229 | — | — | KDIGO |
| Gholoobi et al[[9](#_ENREF_9)] 2015 | 37(73) | 37(73) | 26(51) | NR | NR | NR | NR | NR | — | — | — | — | NR | NR | Scr↑>0.3 mg/dL within 48 h |
| Gallagher et al[[10](#_ENREF_10)] 2015 | 71  (83) | 55  (64) | 67  (78) | 45  (52) | 121.1±22.1 | 121.1±23.9 | 51±8.9 | 51±8.9 | 94±36.3 | 94±30.4 | 58±23.7 | 66±30.4 | — | — | AKIN |
| Candilio et al[[11](#_ENREF_11)] 2015 | 135  (76) | 52  (29) | 132  (74) | 51  (29) | NR | NR | NR | NR | 96.7±32.6 | 89.6±31.0 | 64.8±26.4 | 61.5±26.9 | — | — | AKIN |
| Xu et al[[12](#_ENREF_12)] 2014 | 127  (64) | NR | NR | 46  (23) | 74.4±17.4 | 77.9±16.8 | 100.8±28.2 | 99.1±20.6 | — | — | — | — | 163.3±39.0 | 171.5±37.9 | Scr↑> 25% from the baseline value |
| Savaj et al[[13](#_ENREF_13)] 2014 | 68  (7) | 96  (100) | NR | 16  (2) | 97.2±26.5 | 113.2±35.4 | 94.7±40.2 | 78.2±25.8 | — | — | — | — | 123.8±66.6 | 126.6±77.2 | KDIGO |
| Murphy et al[[14](#_ENREF_14)] 2014 | 36  (58) | 12  (19) | 36  (58) | 11  (18) | 90±17.0 | 86±27.4 | NR | NR | NR | NR | 59±20.7 | 68±22.2 | — | — | AKIN |
| Mouton et al[[15](#_ENREF_15)] 2014 | NR | NR | NR | NR | NR | NR | NR | NR | — | — | — | — | — | — | AKIN |
| Hong et al[[16](#_ENREF_16)] 2014 | 622  (49) | 387  (30) | 689  (54) | NR | NR | NR | NR | NR | 157.6±60.1 | 161.8±65.3 | 97.8±42.7 | 99.7±45.2 | — | — | Scr >2.0 mg/dL or ↑>0.7 mg/dL from the baseline |
| Luo et al[[17](#_ENREF_17)] 2013 | 135  (66) | 57  (28) | NR | 44  (21) | NR | NR | 100±20 | 101±20 | — | — | — | — | 145±41 | 154±46 | Scr↑> 25% or 44.2 mmol/L over the baseline value |
| Igarashi et al[[18](#_ENREF_18)] 2013 | NR | NR | NR | NR | 99.0±15.0 | 101.7±25.6 | 48.9±6.0 | 47.4±9.4 | — | — | — | — | 91.8±39.4 | 92.9±33.2 | L-FABP-based CI-AKI |
| Young et al[[19](#_ENREF_19)] 2012 | NR | NR | NR | 26  (27) | NR | NR | NR | NR | NR | NR | 104.7±40.6 | 117.4±50.3 | — | — | RIFLE |
| Pedersen et al[[20](#_ENREF_20)] 2012 | NR | NR | NR |  | 32±16.3 | 35±15.6 | 114±67.4 | 115±62.2 | 128±54.8 | 131±50.4 | 30±14.8 | 37±13.3 | — | — | RIFLE |
| Lucchinetti et al[[21](#_ENREF_21)] 2012 | 39  (71) | NR | 47  (85) | 23  (42) | 88.0±24.5 | 91.7±15.4 | NR | NR | 94±24 | 109±30 | 63±24 | 74±33 | — | — | NR |
| Er et al[[22](#_ENREF_22)] 2012 | 91  (91) | 64  (64) | 75  (75) | 41  (41) | 143.2±35.4 | 144.1±22.1 | 41.3±11.9 | 40.6±8.7 | **—** | **—** | **—** | **—** | 103±41 | 124±44 | Scr↑> 0.5 mg/dL or 25% over the baseline value |
| Zimmerman et al[[23](#_ENREF_23)] 2011 | 94  (80) | NR | 45  (38) | NR | NR | NR | NR | NR | 113±37 | 115±37 | 68±35 | 73±38 | — | — | AKIN |
| Choi et al[[24](#_ENREF_24)] 2011 | 18  (24) | 5  (7) | NR | NR | 81.3±15.0 | 80.4±14.1 | 78±16 | 79±16 | 145±42 | 132±34 | 108±29 | 98±27 | — | — | AKIN |
| Walsh et al[[25](#_ENREF_25)]  2010 | 28  (70) | 1  (3) | NR | 8  (20) | 88±19.8 | 97±25.9 | 86±25.6 | 71±23.2 | **—** | **—** | 59±21.1 | 58±12.8 | — | — | NR |
| Venugopal et al[[26](#_ENREF_26)] 2010 | 51  (65) | NR | 59  (76) | 18  (23) | 84.2±21 | 84.6±15.7 | NR | NR | 91±23 | 80±17 | 58±29 | 45±16 | — | — | AKIN |
| Thielmann et al[[27](#_ENREF_27)] 2010 | 49  (92) | NR | 45  (85) | 20  (38) | 103.4±13.4 | 99.9±15.9 | 73.5±21.9 | 85.5±22.5 | 110±29 | 109±23 | 76±19 | 71±18 | — | — | NR |
| Rahman et al[[28](#_ENREF_28)] 2010 | 96  (59) | NR | 120  (74) | NR | 96.4±15.9 | 98.1±15.9 | NR | NR | 96±22 | 100±23 | 71±18 | 76±21 | — | — | Scr↑>0.5 mg/dl |
| Hoole et al[[29](#_ENREF_29)] 2009 | 104  (51) | 40  (20) | NR | NR | NR | NR | NR | NR | — | — | — | — | 187.5±74.2 | 196.7±80.1 | Scr↑>25% |
| Ali et al[[30](#_ENREF_30)] 2007 | 47  (57) | 4  (5) | 37  (45) | 21  (26) | 101±23 | 102±35 | NR | NR | — | — | 55±19 | 55±18 | — | — | Peak Scr >177 μmol/L |

Abbreviations: DM, diabetes mellitus; MI, myocardial infarction; KDIGO, kidney disease improving global outcomes; NR, not reported; AKIN, acute kidney injury network; L-FABP, Liver fatty acid binding protein; RIFLE, risk, injury, failure, loss and end-stage kidney.

**References**

1. Pinaud F, Corbeau J-J, Baufreton C, Binuani J-P, De Brux J-L, Fouquet O, Angoulvant D, Furber A, Prunier F: **Remote ischemic preconditioning in aortic valve surgery: Results of a randomized controlled study**. *J Cardiol* 2016, **67**:36-41.

2. Hu Q, Luo W, Huang L, Huang R, Chen R, Gao Y: **Multiorgan protection of remote ischemic perconditioning in valve replacement surgery**. *J Surg Res* 2016, **200**:13-20.

3. Zarbock A, Schmidt C, Van Aken H, Wempe C, Martens S, Zahn PK, Wolf B, Goebel U, Schwer CI, Rosenberger P *et al*: **Effect of Remote Ischemic Preconditioning on Kidney Injury Among High-Risk Patients Undergoing Cardiac Surgery A Randomized Clinical Trial**. *J Am Med Assoc* 2015, **313**:2133-2141.

4. Yamanaka T, Kawai Y, Miyoshi T, Mima T, Takagaki K, Tsukuda S, Kazatani Y, Nakamura K, Ito H: **Remote ischemic preconditioning reduces contrast-induced acute kidney injury in patients with ST-elevation myocardial infarction: A randomized controlled trial**. *Int J Cardiol* 2015, **178**:136-141.

5. Meybohm P, Bein B, Brosteanu O, Cremer J, Gruenewald M, Stoppe C, Coburn M, Schaelte G, Boening A, Niemann B *et al*: **A Multicenter Trial of Remote Ischemic Preconditioning for Heart Surgery**. *New Engl J Med* 2015, **373**:1397-1407.

6. Menting TP, Sterenborg TB, de Waal Y, Donders R, Wever KE, Lemson MS, van der Vliet JA, Wetzels JF, SchultzeKool LJ, Warle MC: **Remote Ischemic Preconditioning To Reduce Contrast-Induced Nephropathy: A Randomized Controlled Trial**. *Eur J Vasc Endovasc* 2015, **50**:527-532.

7. Healy DA, Feeley I, Keogh CJ, Scanlon TG, Hodnett PA, Stack AG, Moloney MC, Whittaker P, Walsh SR: **Remote ischemic conditioning and renal function after contrast-enhanced CT scan: A randomized trial**. *Clinl Invest Med Medicine* 2015, **38**:E110-E118.

8. Hausenloy DJ, Candilio L, Evans R, Ariti C, Jenkins DP, Kolvekar S, Knight R, Kunst G, Laing C, Nicholas J *et al*: **Remote Ischemic Preconditioning and Outcomes of Cardiac Surgery**. *New Engl J Med* 2015, **373**:1408-1417.

9. Gholoobi A, Sajjadi SM, Shabestari MM, Eshraghi A, Shamloo AS: **The Impact of Remote Ischemic Pre-Conditioning on Contrast-Induced Nephropathy in Patients Undergoing Coronary Angiography and Angioplasty: A Double-Blind Randomized Clinical Trial**. *Electron physician* 2015, **7**:1557-1565.

10. Gallagher SM, Jones DA, Kapur A, Wragg A, Harwood SM, Mathur R, Archbold RA, Uppal R, Yaqoob MM: **Remote ischemic preconditioning has a neutral effect on the incidence of kidney injury after coronary artery bypass graft surgery.** *Kidney Int* 2015, **87**: 473-481.

11. Candilio L, Malik A, Ariti C, Barnard M, Salvo C, Lawrence D, Hayward M, Yap J, Roberts N, Sheikh A *et al*: **Effect of remote ischaemic preconditioning on clinical outcomes in patients undergoing cardiac bypass surgery: a randomised controlled clinical trial**. *Heart* 2015, **101**: 185-192.

12. Xu XH, Zhou YJ, Luo SJ, Zhang WJ, Zhao YX, Yu M, Ma Q, Gao F, Shen H, Zhang JW: **Effect of Remote Ischemic Preconditioning in the Elderly Patients With Coronary Artery Disease With Diabetes Mellitus Undergoing Elective Drug-Eluting Stent Implantation**. *Angiology* 2014, **65**:660-666.

13. Savaj S, Savoj J, Jebraili I, Sezavar SH: **Remote ischemic preconditioning for prevention of contrast-induced acute kidney injury in diabetic patients**. IranJ Kidney Dis 2014, 8: 457-460.

14. Murphy N, Vijayan A, Frohlich S, O'Farrell F, Barry M, Sheehan S, Boylan J, Conlon N: **Remote ischemic preconditioning does not affect the incidence of acute kidney injury after elective abdominal aortic aneurysm repair**. *J Cardiothorac Vasc Anesth* 2014,**28**: 1285-1292.

15. Mouton R, Pollock J, Soar J, Mitchell D, Rogers C: **Remote ischaemic preconditioning for elective abdominal aortic aneurysm (AAA) repair: a randomized controlled trial to assess feasibility**. *Appl Cardiopulm Pathophysiol* 2014, **18**: 35.

16. Hong DM, Lee E-H, Kim HJ, Min JJ, Chin J-H, Choi D-K, Bahk J-H, Sim J-Y, Choi I-C, Jeon Y: **Does remote ischaemic preconditioning with postconditioning improve clinical outcomes of patients undergoing cardiac surgery? Remote Ischaemic Preconditioning with Postconditioning Outcome Trial**. *Eur Heart J* 2014, **35**:176-183.

17. Luo SJ, Zhou YJ, Shi DM, Ge HL, Wang JL, Liu RF: **Remote Ischemic Preconditioning Reduces Myocardial Injury in Patients Undergoing Coronary Stent Implantation**. *Can J Cardiol* 2013, **29**:1084-1089.

18. Igarashi G, Iino K, Watanabe H, Ito H: **Remote Ischemic Pre-Conditioning Alleviates Contrast-Induced Acute Kidney Injury in Patients With Moderate Chronic Kidney Disease**. *Circ J* 2013, **77**:3037-3044.

19. Young PJ, Dalley P, Garden A, Horrocks C, La Flamme A, Mahon B, Miller J, Pilcher J, Weatherall M, Williams J *et al*: **A pilot study investigating the effects of remote ischemic preconditioning in high-risk cardiac surgery using a randomised controlled double-blind protocol**. *Basic Res Cardiol* 2012, **107**:256.

20. Pedersen KR, Ravn HB, Povlsen JV, Schmidt MR, Erlandsen EJ, Hjortdal VE: **Failure of remote ischemic preconditioning to reduce the risk of postoperative acute kidney injury in children undergoing operation for complex congenital heart disease: A randomized single-center study**. *J Thorac Cardiov Sur* 2012, **143**:576-583.

21. Lucchinetti E, Bestmann L, Feng JH, Freidank H, Clanachan AS, Finegan BA, Zaugg M: **Remote Ischemic Preconditioning Applied during Isoflurane Inhalation Provides No Benefit to the Myocardium of Patients Undergoing On-pump Coronary Artery Bypass Graft Surgery Lack of Synergy or Evidence of Antagonism in Cardioprotection?** *Anesthesiology* 2012, **116**:296-310.

22. Er F, Nia AM, Dopp H, Hellmich M, Dahlem KM, Caglayan E, Kubacki T, Benzing T, Erdmann E, Burst V *et al*: **Ischemic Preconditioning for Prevention of Contrast Medium-Induced Nephropathy Randomized Pilot RenPro Trial (Renal Protection Trial)**. *Circulation* 2012, **126**:296-303.

23. Zimmerman RF, Ezeanuna PU, Kane JC, Cleland CD, Kempananjappa TJ, Lucas FL, Kramer RS: **Ischemic preconditioning at a remote site prevents acute kidney injury in patients following cardiac surgery**. *Kidney Int* 2011, **80**:861-867.

24. Choi YS, Shim JK, Kim JC, Kang KS, Seo YH, Ahn KR, Kwak YL: **Effect of remote ischemic preconditioning on renal dysfunction after complex valvular heart surgery: a randomized controlled trial**. *J Thorac Cardiov Sur* 2011, **142:** 148-154.

25. Walsh SR, Sadat U, Boyle JR, Tang TY, Lapsley M, Norden AG, Gaunt ME: **Remote Ischemic Preconditioning for Renal Protection During Elective Open Infrarenal Abdominal Aortic Aneurysm Repair: Randomized Controlled Trial**. *Vasc Endovasc Surg* 2010, **44**:334-340.

26. Venugopal V, Laing CM, Ludman A, Yellon DM, Hausenloy D: **Effect of Remote Ischemic Preconditioning on Acute Kidney Injury in Nondiabetic Patients Undergoing Coronary Artery Bypass Graft Surgery: A Secondary Analysis of 2 Small Randomized Trials**. *Am J Kidney Dis* 2010, **56**:1043-1049.

27. Thielmann M, Kottenberg E, Boengler K, Raffelsieper C, Neuhaeuser M, Peters J, Jakob H, Heusch G: **Remote ischemic preconditioning reduces myocardial injury after coronary artery bypass surgery with crystalloid cardioplegic arrest**. *Basic Res Cardiol* 2010, **105**:657-664.

28. Rahman IA, Mascaro JG, Steeds RP, Frenneaux MP, Nightingale P, Gosling P, Townsend P, Townend JN, Green D, Bonser RS: **Remote ischemic preconditioning in human coronary artery bypass surgery: from promise to disappointment?** *Circulation* 2010, **122**: S53-59

29. Hoole SP, Heck PM, Sharples L, Khan SN, Duehmke R, Densem CG, Clarke SC, Shapiro LM, Schofield PM, O'Sullivan M *et al*: **Cardiac Remote Ischemic Preconditioning in Coronary Stenting (CRISP Stent) Study A Prospective, Randomized Control Trial**. *Circulation* 2009, **119**:820-827.

30. Ali ZA, Callaghan CJ, Lim E, Ali AA, Nouraei SAR, Akthar AM, Boyle JR, Varty K, Kharbanda RK, Dutka DP *et al*: **Remote ischemic preconditioning reduces myocardial and renal injury after elective abdominal aortic aneurysm repair - A randomized controlled trial**. *Circulation* 2007, **116**:I98-I105.
